# Status Threat or Grateful Reciprocity? Investigating Pathways Through Which Receiving Task-Related Help Influences Recipients’ Subsequent Differentiated Helping Behavior

**DOI:** 10.3390/bs16071205

**Published:** 2026-07-17

**Authors:** Huabin Wu, Jiyuan Chen, Binlin Si, Lijun Xia, Yu Mao

**Affiliations:** 1 School of Entrepreneurship, Zhejiang University of Finance and Economics Dongfang College, Haining 314408, China; 2 School of Business, Nanjing University, Nanjing 210008, China

**Keywords:** task-related help, status threat, dependency-oriented help, autonomy-oriented help, competitive climate, behavioral strategy choice

## Abstract

Employees’ helping behavior is critical for team efficiency and innovation. However, the literature highlights competing perspectives on whether receiving help elicits a pattern consistent with reciprocity or defensive ingratitude. Grounded in the help-as-status model and self-regulation theory, we developed a dual-pathway model to examine how received task-related help may influence recipients’ subsequent differentiated helping behaviors through the lens of status threats. We further investigate the moderating role of a competitive climate in this process. On the basis of a two-wave survey of 252 full-time employees, our findings reveal that receiving task-related help is positively associated with perceived status threat. This threat, in turn, serves as a plausible mediator of the link between receiving help and subsequent dependency-oriented help while exerting a suppressive effect on autonomy-oriented help. Furthermore, a competitive climate appeared to intensify the indirect pathway to dependency-oriented help, though the evidence for moderation of the autonomy-oriented help pathway was mixed. By exploring these defensive psychological mechanisms within a single national organizational context, this study offers a cautious, status-based explanation for managing the complexities of prosocial interactions. While the findings provide nuanced insights into post-help dynamics, we acknowledge the inherent boundaries of our two-wave, self-reported survey design. However, the positive direct association with autonomy-oriented help may be consistent with multiple processes beyond status threat, including unmeasured gratitude, reciprocity norms, learning orientation, or enhanced relational quality.

## 1. Introduction

Employee helping behavior is foundational for building interpersonal trust, enhancing team efficiency, and driving organizational innovation (Siemsen et al., 2007). By facilitating knowledge exchange and skill transfer, mutual assistance enables individuals to accumulate human capital and helps organizations navigate volatile environments (Shah et al., 2015). Consequently, helping is widely regarded as a critical prosocial catalyst for positive organizational culture and sustainable competitive advantage (Mueller & Kamdar, 2011). However, the theoretical landscape of post-help interactions is increasingly recognized as being complex and non-linear. Traditional perspectives emphasize an inherently prosocial narrative, asserting that receiving help stimulates generalized reciprocity and motivates recipients to assist their benefactors or other team members (Bommer et al., 2003; Chiaburu & Harrison, 2008).

Recently, this uniformly positive assumption has been challenged. Emerging research reveals that receiving help can paradoxically threaten a recipient’s self-esteem, elicit envy (Tai et al., 2023), and suppress subsequent citizenship behavior (Y. E. Lee et al., 2023). This simplification overlooks the nuanced dimensions of how assistance is provided. Specifically, we move beyond this binary narrative by distinguishing between dependency-oriented and autonomy-oriented help as functional responses. Unlike existing research that often views reciprocity as a uniformly prosocial outcome, our status-based perspective uncovers the logic of “defensive prosociality”—defined as a behavioral pattern of providing help that is functionally consistent with status-defense, without implying conscious strategic intent—which corresponds to the prevention focus in self-regulation theory, explaining how help recipients navigate the tension between maintaining social bonds and maintaining their relative standing. To explain why individuals adopt these specific pathways, we integrate the help-as-status model with self-regulation theory. We posit that perceived status threat serves as the critical psychological “missing link” that explains the shift from communal to competitive logic following a task-related help interaction. Ultimately, our parallel mediation framework elucidates the psychological conditions under which receiving help motivates functional and status-defensive helping behaviors, thereby offering a potential explanation for prior inconsistent findings in the prosociality literature.

Specifically, we distinguish between dependency-oriented (dependent) and autonomy-oriented (autonomous) forms of help. Dependency-oriented help involves directly resolving an immediate problem for a recipient without fostering independent problem-solving capabilities (Nadler, 2002). In contrast, autonomy-oriented help involves transferring requisite skills and knowledge, empowering the recipient to independently address similar challenges in the future. Dependency-oriented help often yields suboptimal long-term effects (Alvarez & van Leeuwen, 2014) and can be functionally deployed as a tool for status competition rather than as genuine support (Nadler & Chernyak-Hai, 2014). Because it maintains a prosocial facade while structurally reinforcing dependency, we conceptualized receiving help followed by providing help as a functionally ambivalent behavioral pathway.

To explain why individuals adopt this specific pathway, we integrate the help-as-status model with self-regulation theory. The help-as-status model posits that helping interactions inherently reflect and reinforce status hierarchies (Nadler, 2002; Nadler et al., 2009). Accordingly, receiving task-related help, which directly involves core professional competencies, signals relative incompetence, thereby triggering a perceived status threat (Bunderson, 2003; Cheng et al., 2013). We explicitly focus on task-related help because, unlike emotional support, it directly challenges a recipient’s perceived work ability (Mathieu et al., 2019). While negative affective states often prompt withdrawal, self-regulation theory suggests that individuals actively recalibrate their behavior to bridge the gap between their threatened standing and their goal of maintaining a high status (Bandura, 1991; Lord et al., 2010). We posit that recipients mitigate this status threat by functionally providing dependency-oriented help, signalling their own competence without empowering others, while being more likely to suppress autonomy-oriented help to safeguard their relative positions.

Furthermore, acknowledging that self-regulation is context-dependent (Bandura, 1991), we identified a competitive climate as a crucial boundary condition. In highly competitive environments, employees prioritize their relative standing and outperform their peers (David et al., 2021). This context amplifies sensitivity to the competence-threatening signals of receiving help, thereby intensifying the perceived status threat (Nadler et al., 2009). Ultimately, our parallel mediation framework elucidates the psychological conditions under which receiving help motivates functional and status-defensive helping behavior. In doing so, we extend the prosociality literature and provide actionable insights for managing team dynamics in competitive organizational settings.

The remainder of this article is organized as follows. First, we review the relevant literature and integrate the help-as-status model with self-regulation theory to develop our hypotheses regarding the dual mediated pathways. Next, we introduce competitive climate and formulate our moderation hypotheses. We then describe our research methodology, including the sample, procedures of the two-wave survey, and measures, followed by the presentation of our empirical results. Finally, we discuss the theoretical and practical implications of our findings, acknowledge the limitations of the current study, and outline promising avenues for future research.

## 2. Theoretical Background and Hypotheses

### 2.1. The Help-as-Status Perspective and Perceived Status Threat

Organizational status represents an individual’s relative standing within a social hierarchy and is characterized by the level of respect, prominence, and influence afforded by peers (Djurdjevic et al., 2017). A primary antecedent of status is the demonstration of task competence (Bunderson, 2003). According to the help-as-status model, interpersonal helping is not merely a prosocial exchange but a status-laden interaction that inherently signals the helper’s superiority (Nadler & Chernyak-Hai, 2014). Receiving task-related help—assistance explicitly directed at core performance objectives (Deelstra et al., 2003)—forcefully highlights the recipient’s inability to independently address the challenges. This dynamic invites peer scrutiny of the recipient’s professional viability (Schroeder et al., 2015), thereby triggering a profound perceived status threat. It should be noted that status threat differs from competence threat and self-esteem threat. Status threat reflects an individual’s perception that their relative rank and prestige within the team may decline, with its focus being relational and hierarchical. In contrast, competence threat refers to an individual’s perception of poor performance on a specific task, while self-esteem threat refers to an individual’s perception that their overall self-worth is being negated. These three constructs are fundamentally distinct from one another.

Unlike emotional support, which affects psychological well-being, task-related help directly affects a recipient’s core work ability (Bamberger et al., 2017). It is crucial to distinguish perceived status threat from conceptually related constructs such as self-esteem, competence, and resource threats. While self-esteem threat involves a blow to one’s global self-worth and competence threat focuses specifically on the doubt regarding one’s ability to perform a task, status threat is uniquely relational and hierarchical. It represents an individual’s concern over their relative standing, respect, and influence within a social collective. Unlike resource threat, which centers on the depletion of personal assets, status threat highlights the social-structural costs of interpersonal interactions. In the context of receiving help, status threat captures the recipient’s anxiety that the help signals a lower informal rank, thereby inviting peer scrutiny of their long-term professional viability rather than just a temporary skill deficit. Needing assistance suggests a resource or skill deficit, creating a stark contrast between the helper’s abundance and the recipient’s dependency (Alvarez & van Leeuwen, 2011). Frequent reliance on such help implicitly signals a lower potential for future promotions and diminishes the social standing within the team hierarchy. Thus, while instrumentally beneficial, task-related help functions as a competence-threatening signal that undermines the recipient’s perceived status.

**Hypothesis 1.**  *Task-related help is positively associated with perceived status threat.*

### 2.2. Self-Regulation and Differentiated Helping Responses

How do individuals respond to the psychological tension caused by status threats? While traditional perspectives might predict withdrawal, self-regulation theory posits that individuals are proactive agents who calibrate their behaviors to minimize the discrepancy between their threatened current state and their desired high-status goal (Bandura, 1991; Lord et al., 2010). Driven by an internal psychological need to restore a sense of competence and generosity (Carson Marr et al., 2019), threatened individuals are more likely to gravitate toward behaviors that reaffirm their value to the collective group. Providing help serves as a key mechanism for facilitating status restoration (Flynn et al., 2006).

However, not all forms of help serve the same self-regulatory functions. Dependency-oriented (dependent) help involves resolving a peer’s immediate problem without transferring the underlying skills needed for future independence (Nadler, 2002). For a status-threatened individual, dependency-oriented help offers a functional, albeit ambivalent, advantage: it provides a visible signal of “helpful competence” while having the practical effect of keeping the recipient structurally reliant on the helper (Nadler & Chernyak-Hai, 2014). This behavioral tendency allows threatened individuals to restore their image of competence without eroding their unique informational advantage ( Carson Marr et al., 2019).

Conversely, autonomy-oriented (autonomous) help, which focuses on comprehensive skill transfer and empowers the recipient to be completely self-sufficient—represents a highly risky self-regulatory prospect under conditions of status threat. According to self-regulation theory, when individuals experience a discrepancy between their threatened current state and desired status, they become highly vigilant about resource conservation and their relative irreplaceability (Bandura, 1991). Providing autonomy-oriented help inherently involves relinquishing one’s monopoly over critical expertise. By equipping peers with independent problem-solving capabilities, helpers effectively dilute their unique informational advantage, making themselves less indispensable to the team’s future operations (Bamberger et al., 2017). For an employee actively navigating a status threat, empowering a peer is cognitively processed as transferring the very “competence capital” needed to secure their own hierarchical standing. Therefore, individuals experiencing status threats tend to become less inclined to provide autonomy-oriented help, a behavioral pattern consistent with safeguarding competitive standing.

**Hypothesis 2a.**  *Perceived status threat is positively associated with the provision of dependency-oriented help.*

**Hypothesis 2b.**  *Perceived status threat is negatively associated with the provision of autonomy-oriented help.*

### 2.3. Mediating Role of Perceived Status Threat

Integrating these arguments, we propose that perceived status threat serves as the critical psychological mechanism through which receiving task-related help dictates subsequent differentiated-helping responses. Receiving task-related assistance predicts an acute appraisal of competence failure and potential hierarchical demotion (Deelstra et al., 2003). Driven by the self-regulatory goal to bridge this status discrepancy, recipients often exhibit a pattern of “defensive prosociality” (Chernyak-Hai & Davidai, 2022). This response is inherently dual-faceted in nature. Proactively, threatened individuals may gravitate toward providing dependency-oriented help to signal utility and foster a sense of reciprocal obligation without genuinely elevating their peers’ capabilities (Nadler et al., 2009). On the restrictive side, heightened anxiety regarding their precarious relative position diminishes their willingness to offer autonomy-oriented help. Because transferring core skills further erodes their unique value to the organization, the self-regulatory response often manifests as a reluctance to empower others to prevent any further loss of competitive advantage.

**Hypothesis 3a.**  *Perceived status threat mediates the positive relationship between receiving task-related help and providing dependency-oriented help.*

**Hypothesis 3b.**  *Perceived status threat mediates the negative relationship between receiving task-related help and providing autonomy-oriented help.*

### 2.4. The Moderating Role of a Competitive Climate

The psychological impact of receiving help is significantly contingent upon the organizational context. Self-regulation theory emphasizes that external environments dictate which goals, prosocial or ego-defensive, become salient (Bandura, 1991). We identify a psychologically competitive climate as a crucial boundary condition. Psychological climate refers to an individual’s subjective perception and cognitive appraisal of their work environment. It is necessary to clarify that this study adopts individual-level psychological competitive climate. The specific reasons are as follows. First, this study adopts the perspective of self-regulation theory and the status model. According to self-regulation theory, our focus is on psychological climate rather than group-level aggregated climate, as this theory emphasizes that individuals’ behaviors are driven by their subjective interpretations of the external environment. Moreover, the status model also reflects individuals’ perception of their own relative standing. Second, this study focuses on psychological mechanisms. Our model examines how recipients perceive status threat and provide different types of help after receiving task-related help, which is consistent with the theoretical model’s level of analysis. Third, within the same team and facing the same objective competitive environment, different individuals may experience different levels of threat perception and subsequent behavioral responses due to factors such as their status sensitivity. Using individual-level perceived competition as a moderator can reveal these differentiated psychological processes.

Because self-regulatory behaviors are driven by how individuals personally interpret situational cues, the perceived intensity of interpersonal competition is the most direct contextual moderator of their psychological threat and subsequent behavior. When individuals perceive a highly competitive psychological climate, they are more likely to adopt a zero-sum belief system and engage in frequent upward social comparisons. Under these conditions, organizational status and resources are viewed as strictly finite, meaning that one employee’s demonstration of competence is cognitively processed as a direct loss to another’s relative standing (David et al., 2021). Consequently, the status-signalling nature of helping is profoundly intensified. Receiving help is no longer interpreted as a benign communal exchange but is instead scrutinized through the lens of zero-sum competition. In this context, the need for assistance serves as a glaring signal of competitive disadvantage and resource scarcity, which significantly magnifies the psychological experience of status threat (Hughes et al., 2024). In contrast, in low-competition environments, help is more likely to be appraised as a communal resource, attenuating the threat to one’s professional standing.

**Hypothesis 4.**  *A competitive climate positively moderates the relationship between receiving task-related help and perceived status threats.*

Finally, combining the aforementioned arguments, we propose a moderated mediation framework. In a highly competitive climate, organizational status and resources are perceived as strictly zero-sum games. Under these conditions, the competence-threatening signals inherent in receiving task-related help are profoundly magnified, associated with an intensified experience of status threats. This amplified psychological threat, in turn, heavily dictates the recipient’s self-regulatory resource allocation. Specifically, in highly competitive environments, the indirect effect of receiving help on the adoption of defensive-helping tendencies becomes substantially stronger. Threatened individuals exhibit a more pronounced behavioral shift: they are more likely to rely on dependency-oriented help, a pattern consistent with competence-signaling, while simultaneously exhibiting a stronger reluctance to engage in autonomy-oriented help, a pattern consistent with the preservation of relative status advantages.

**Hypothesis 5a.**  *A competitive climate moderates the indirect effect of receiving task-related help on dependency-oriented help through perceived status threat.*

**Hypothesis 5b.**  *A competitive climate moderates the indirect effect of receiving task-related help on autonomy-oriented help through perceived status threat.*

**Figure 1:**
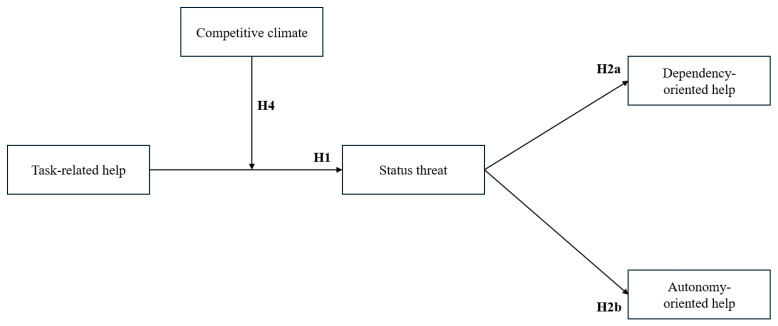
Research Model. Note: H1, H2a, H2b, and H4 represent direct and moderating paths, respectively, as indicated. H3a and H3b (not shown on paths) predict the mediating role of perceived status threat. H5a and H5b predict the moderated mediation effects of a competitive climate.

We construct the research model as shown in Figure 1.

## 3. Research Design

### 3.1. Sample and Procedure

To test our hypotheses, we conducted a two-wave, time-lagged survey of full-time employees in China via Credamo (Credamo Inc., Beijing, China), a professional primary data provider widely recognized in management research for its high-quality participant pool. The participants recruited through this platform were independent employees dispersed across a wide range of organizations and industries nationwide. Because these respondents were not nested within a single organizational unit or specific work team, our data structure did not exhibit clustering effects, making standard single-level analytical techniques appropriate. This multi-wave design was specifically chosen to reduce the risk of potential common method bias (CMB) by creating a temporal separation between the measurement of the independent and dependent variables (Podsakoff et al., 2003). A one-week interval was deemed appropriate because it provides sufficient temporal distance to reduce the transient mood states and consistency motifs typically found in one-shot surveys while remaining short enough to accurately capture the relatively immediate psychological and behavioral reactions to specific workplace interactions before they are confounded by subsequent organizational events. However, we acknowledge that the one-week interval is relatively short. Because all focal variables were ultimately self-reported by the same respondents, procedural remedies could not entirely eliminate the risk of percept–percept inflation and CMB.

To proactively minimize the influence of common method bias through survey design, we implemented several procedural remedies in this study. Beyond the temporal separation of the two survey waves, we assured participants of strict anonymity and confidentiality and explicitly emphasized that there were no right or wrong answers to reduce evaluation apprehension and social desirability bias. At Time 1, participants provided demographic information and evaluated the task-related help they received, perceived status threat, and the psychological competitive climate they perceived within their teams. We also embedded attention-check items to ensure data quality. One week later (Time 2), participants who passed the initial screening were invited to report their subsequent helping behaviors (dependency- and autonomy-oriented help) provided to their colleagues.

Of the 300 initial invitations, 252 valid matched responses were obtained (85% effective response rate). To ensure data integrity, we excluded surveys with completion times shorter than the threshold (180 s) or those that exhibited patterned responses. The final sample was diverse: 67% were female, more than 90% held a bachelor’s degree or higher, and the majority (approximately 90%) were aged between 20 and 40 years. The participants represented various industries and team sizes, with 40% working in teams of three to nine members, providing a robust context for observing interpersonal-helping dynamics.

### 3.2. Measures

All the measures were derived from established scales and translated into Chinese following standard back-translation procedures (Brislin, 1970) to ensure linguistic equivalence. The detailed measurement items are provided in Appendix A. Participants responded using a 7-point Likert scale (1 = “strongly disagree,” 7 = “strongly agree”).

(1) Task-related help received (Time 1). We assessed this construct using a 4-item scale developed by Settoon and Mossholder (2002) and recently validated in post-help contexts by Tai et al. (2023). A sample item is “My colleagues take time to help me with task-related problems.” The Cronbach’s alpha was 0.879.
(2) Perceived status threat (Time 1). We used a 3-item scale from Okimoto and Wenzel (2011). Although concise, this scale is highly appropriate for our model because it precisely captures the unidimensional, relational essence of status threat—specifically, an individual’s acute anxiety regarding the loss of respect, influence, and relative standing within a group. Furthermore, the use of a focused and well-validated short scale allowed us to accurately isolate this specific psychological reaction while effectively minimizing respondent fatigue and cognitive load in a multi-wave survey. A sample item was “I feel that my professional standing within the team is being challenged.” Cronbach’s alpha was 0.771.
(3) Competitive climate (Time 1). We utilized the 4-item scale from Brown et al. (1998) to assess the psychological competitive climate, specifically capturing the degree of perceived interpersonal competition at the individual level. A sample item was “My manager frequently compares my performance with that of my colleagues.” The Cronbach’s alpha was 0.786.
(4) Dependency- and autonomy-oriented help (Time 2). To capture differentiated helping behaviors, we employed a 10-item scale (five items per dimension) developed by Koopmann (2016). This specific scale was selected because it perfectly aligns with our theoretical focus on “defensive prosociality” and the preservation of an “expertise monopoly”. Unlike broader, generic measures of organizational citizenship behavior (OCB), Koopmann’s (2016) scale was explicitly developed for a professional workplace context. It meticulously captures the behavioral dichotomy between transferring underlying capabilities (autonomy-oriented help) and merely providing immediate, dependency-inducing solutions (dependency-oriented help). Therefore, it uniquely operationalizes the exact functional and behavioral distinctions central to our status-defense framework. Sample items are “I provided direct solutions to colleagues’ problems without explaining the underlying logic” (the Cronbach’s alpha of dependency-oriented help = 0.878) and “I taught colleagues the skills necessary to solve similar problems independently in the future” (the Cronbach’s alpha of autonomy-oriented help = 0.817).
(5) Control Variables. We controlled for demographic factors that potentially influence helping tendencies, including gender, age, education, and tenure. We also controlled for team size, as the visibility of status and the necessity of help often vary with the scale of the social group.

## 4. Empirical Analysis

### 4.1. Confirmatory Factor Analysis

To evaluate the construct validity and distinctiveness of the focal variables, we conducted a series of confirmatory factor analyses (CFAs). As summarized in Table 1, the hypothesized five-factor model demonstrated a superior fit to the data (χ^2^/df = 1.949, CFI = 0.924, TLI = 0.911, RMSEA = 0.061, SRMR = 0.056). This baseline model yielded significantly better fit indices than any of the alternative nested models did (e.g., combining dependent and autonomy-oriented help into a single factor). These results provide supportive evidence of the discriminant validity of our study variables, confirming that they represent distinct theoretical and empirical constructs.

### 4.2. Common Method Bias Test

While we initially performed Harman’s single-factor test as a preliminary diagnostic tool, we acknowledge its limitations as a minimal-assessment tool. The results indicated that the first unrotated factor accounted for only 20.99% of the total variance, which was well below the conventional 40% threshold. Therefore, to rigorously evaluate the potential threat of common method bias and ensure the robustness of our findings, we relied primarily on an unmeasured latent method factor approach. Specifically, we specified a model wherein all self-reported items were allowed to load simultaneously on their hypothesized theoretical constructs as well as on an orthogonal, unmeasured method factor. The inclusion of the method factor yielded improvements in fit indices (χ^2^(158) ≈ 241.9, χ^2^/df ≈ 1.531, CFI = 0.963, TLI = 0.949, RMSEA = 0.046, SRMR = 0.043), corresponding to changes relative to the five-factor baseline of ΔCFI = 0.039, ΔTLI = 0.038, ΔRMSEA = 0.015, and ΔSRMR = 0.013. While these improvements suggest that some method variance is present, it is important to note that the substantive factor loadings on their hypothesized constructs, as well as the significance of our hypothesized structural paths, remained robust and virtually unchanged after controlling for this method factor. Therefore, while common method bias cannot be ruled out entirely given the self-reported nature of our data, it is unlikely to be the primary confound that fundamentally distorts our inferences.

### 4.3. Descriptive Statistics and Correlation Analysis

As shown in Table 2, the composite reliability (CR) for all the variables remained robust, exceeding 0.80, and most standardized factor loadings were above 0.70, indicating adequate internal consistency. The average variance extracted (AVE) values for task-related help, perceived status threat, and dependency-oriented help were 0.646, 0.529, and 0.591, respectively, all exceeding the conventional 0.50 threshold.

However, we must acknowledge that the AVEs for autonomy-oriented help (0.480) and a competitive climate (0.486) fell slightly below this benchmark. While their robust CR values and factor loadings provide acceptable support for convergent validity as a conservative estimate (Lam, 2012), we interpret these specific measures cautiously, recognizing that these two constructs were measured less clearly than is ideal in this sample.

With respect to discriminant validity, as summarized in Table 3, the square root of the AVE for each construct was strictly greater than the highest correlation with any other latent variable. Furthermore, all the Pearson correlation coefficients remained below 0.50, suggesting that the study variables maintained sufficient empirical distinctiveness. Specifically, we explicitly acknowledge that the AVE values for autonomy-oriented help and competitive climate fell slightly below the preferred 0.50 threshold. This suggests that these two constructs were measured less cleanly than ideal in this sample, and as such, their corresponding effects should be interpreted with appropriate caution.

### 4.4. Hypothesis Testing

#### 4.4.1. Impact of Receiving Task-Related Help on Perceived Status Threat

We first examined the relationship between receiving task-related help and perceived status threat. As reported in Table 4, after accounting for all control variables, received task-related help was significantly and positively associated with perceived status threat (B = 0.163, SE = 0.075, *p* < 0.05). The inclusion of this focal predictor in Model 2 accounted for significant incremental variance beyond the control-only model (ΔR^2^ = 0.03), providing empirical support for Hypothesis 1. Notably, the results also revealed a significant negative association between work tenure and perceived status threat (B = −0.379, SE = 0.129, *p* < 0.01). These findings are consistent with the perspective that organizational tenure serves as a proxy for seniority and human capital accumulation; individuals with longer tenure typically have higher informal vertical status and possess greater “insider status” (Z. Liu et al., 2024). Consequently, such individuals are likely to experience greater status security, which acts as a psychological buffer against the competence-threatening signals inherent in receiving help. This observed effect of tenure further underscores the intrinsic and status-laden nature of interpersonal helping interactions within professional hierarchies in the workplace.

#### 4.4.2. Testing the Mediating Role of Perceived Status Threat

To examine the mediating role of perceived status threat, we followed a rigorous multistep analytical procedure consistent with established methodological recommendations. First, we estimated the total effect of the received task-related help on the two helping behaviors. Second, we verified the relationship between the independent variable and our hypothesized mediator, perceived status threat. Third, we assessed the direct impact of perceived status threat on subsequent helping behaviors. Finally, we specified a simultaneous path model incorporating independent, mediating, and control variables to determine the nature of the mediation. A reduction in the significance or magnitude of the direct path from receiving task-related help to subsequent behaviors upon the inclusion of perceived status threat would provide preliminary evidence of mediation. To ensure the robustness of these findings, we employed Mplus 8.3 (Muthén & Muthén, Los Angeles, CA, USA) to conduct a bootstrapping analysis that generated more accurate confidence intervals for indirect effects.

(1) Mediating role of perceived status threat between receiving task-related help and subsequent dependency-oriented help.

To test whether perceived status threat mediates the link between receiving task-related help and receiving dependency-oriented help, we first estimated the baseline relationship. As shown in Table 5 (Model 2), task-related help received was significantly and positively associated with subsequent dependency-oriented help (B = 0.154, SE = 0.070, *p* < 0.05), providing preliminary support for the overall mediation framework. Subsequently, when perceived status threat was entered as the mediator (Model 3), it demonstrated a significant positive association with dependency-oriented help (B = 0.471, SE = 0.065, *p* < 0.001), supporting Hypothesis 2a. Upon entering the mediator in Model 3, perceived status threat exhibited a strong positive association with dependency-oriented help (B = 0.471, SE = 0.065, *p* < 0.001), yielding a substantial increase in the explained variance (ΔR^2^ = 0.179, *p* < 0.001). In the final integrated model (Model 4), while the effect of perceived status threat remained robust (B = 0.460, SE = 0.069, *p* < 0.001), the previously significant path from received task-related help to dependency-oriented help became non-significant (B = 0.078, SE = 0.069, ns). This pattern provides preliminary evidence that the relationship is statistically accounted for by the recipient’s perceived status threat, lending initial support to Hypothesis 3a.

We further substantiated the indirect effect using the bootstrapping method with 5000 resamples. As summarized in Table 6, the results revealed a significant indirect effect of the received task-related help on dependency-oriented help via perceived status threat (indirect effect = 0.081), with a 95% bias-corrected confidence interval (CI) that did not straddle zero [95% CI: 0.008, 0.179]. In contrast, the direct effect was non-significant (direct effect = 0.078; 95% CI: [−0.057, 0.208]). The exclusion of zero from the indirect effect interval, coupled with the inclusion of zero in the direct effect interval, is consistent with a mediating role of perceived status threat in this relationship. However, mindful of the observational nature of our self-report survey design, we cautiously interpret that perceived status threat serves as a primary explanatory mechanism linking receiving task-related help and providing dependency-oriented help in the following way. These findings support Hypothesis 3a.

(2) The mediating role of perceived status threat between receiving task-related help and subsequent autonomy-oriented help.

Following the analysis of dependency-oriented help, we examined the relationships among task-related help received, perceived status threat, and autonomy-oriented help received. As reported in Table 7 (Model 2), task-related help was strongly positively associated with subsequent autonomy-oriented help (B = 0.390, SE = 0.065, *p* < 0.001). Upon introducing perceived status threat in Model 3, its relationship with autonomy-oriented help did not reach statistical significance (B = −0.092, SE = 0.080, ns). However, in the complete model (Model 4) that included both received help and status threat, status threat emerged as a significant negative predictor of autonomy-oriented help (B = −0.183, SE = 0.079, *p* < 0.05). H2b was therefore supported in the complete model, as the hypothesized negative association became significant once the positive direct effect of received help was controlled. H3b received qualified support insofar as the hypothesized negative indirect effect was significant (indirect effect = −0.031, 95% CI [−0.100, −0.001]); however, the total positive association between received help and autonomy-oriented help remained positive, indicating inconsistent mediation rather than an overall negative relationship. If the wording of H3b is interpreted as requiring a negative total relationship, Hypothesis 3b may instead be labelled partially supported.

To explore this effect, we employed the bootstrapping method with 5000 resamples. As summarized in Table 8, the indirect effect was significant and negative (indirect effect = −0.031), with a 95% bias-corrected confidence interval (CI) that excluded zero [−0.100, −0.001]. The direct effect remained positive and significant (direct effect = 0.423; 95% CI [0.290, 0.538]). This configuration of results suggests that perceived status threat functions as a psychological suppressor, partially offsetting the positive behavioral outcomes typically elicited by receiving help. Inconsistent mediation occurs when the direct and indirect effects operate in opposite directions, causing the total effect to be smaller in magnitude than the direct effect. In the present case, receiving help simultaneously activates a communal reciprocity logic (driving the positive direct path) and a status-defensive logic (driving the negative indirect path through status threat). Importantly, we explicitly acknowledge that our proposed status-based perspective represents one plausible mechanism rather than the sole psychological process at play. The strong positive direct association between receiving task-related help and providing autonomy-oriented help likely reflects unmeasured alternative explanations, such as pervasive reciprocity norms, the activation of a learning orientation among recipients, or enhanced relational quality between peers. Thus, while receiving help generally encourages autonomous assistance through these broader communal or developmental pathways, the concurrent activation of status-based anxiety serves as a specific, opposing mechanism that drives this pro-social tendency.

#### 4.4.3. Moderating Role of a Competitive Climate

(1) Moderating effect of a competitive climate on the positive impact of receiving task-related help on perceived status threat.

To evaluate the moderating influence of competitive climate, we first estimated a baseline model incorporating both task-related help received and the moderator, along with control variables. As summarized in Table 9 (Model 3), both received task-related help (B = 0.224, SE = 0.072, *p* < 0.01) and a competitive climate (B = 0.408, SE = 0.072, *p* < 0.001) were significantly and positively associated with perceived status threat. Compared with Model 2 (ΔR^2^ = 0.242), the inclusion of a competitive climate yielded a substantial increase in the explained variance, underscoring its relevance as a critical contextual predictor. Subsequently, we introduced the interaction term between the received task-related help and the competitive climate into the model. The results of Model 4 demonstrate that the interaction effect was positive and statistically significant (B = 0.135, SE = 0.069, *p* < 0.05). These findings suggest that a competitive climate reinforces the positive association between receiving task-related help and perceived status threat, providing initial empirical support for Hypothesis 4.

**Figure 2:**
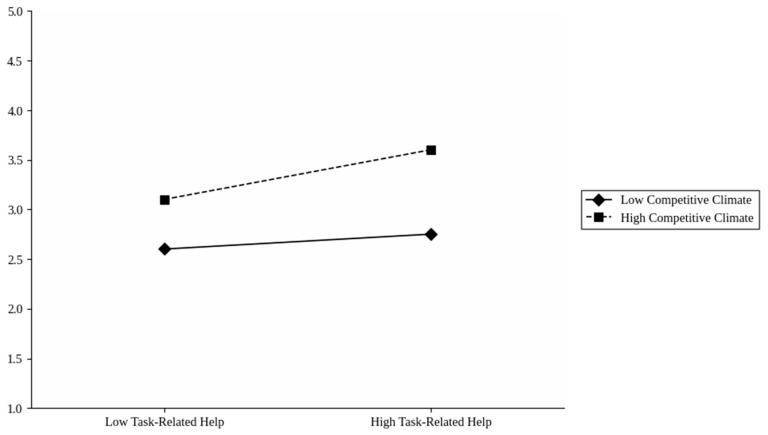
Moderating effect of a competitive climate on the relationship between receiving task-related help and perceived status threat.

Following the identification of a significant interaction effect, we conducted simple slope analyses to further probe the nature of the moderation. Consistent with our theoretical framework, the positive relationship between received task-related help and perceived status threat remained significant in a highly competitive climate (B = 0.242, SE = 0.084, *p* < 0.05). Conversely, this relationship was attenuated and became non-significant when the competitive climate was low (B = 0.065, SE = 0.063, ns). Visual inspection of the interaction plot (Figure 2) further corroborates this pattern, indicating that the status-threatening nature of receiving assistance is contingent on the intensity of interpersonal competition within the team. Collectively, these results provide empirical support for Hypothesis 4, suggesting that a competitive climate acts as a pivotal boundary-intensifying factor in the help-as-status process. However, because both the independent variable and the moderator were self-reported within the same general perceptual environment, we cautiously interpret these moderation effects, acknowledging that shared method variance may partially inflate these relationships.

(2) Moderated Mediation Test

Building on the established link between perceived status threat and subsequent helping behaviors, we evaluated the potential moderated mediation effects for both dependency-oriented help (H5a) and autonomy-oriented help (H5b). We utilized the bootstrapping method with 5000 resamples to estimate conditional indirect effects at low (−1 SD), mean, and high (+1 SD) levels of competitive climate, as well as the index of moderated mediation, which provides a formal test of whether the conditional indirect effects differ significantly across levels of the moderator.

As summarized in Table 10, for dependency-oriented help (H5a), the conditional indirect effects were significant at the mean (B = 0.129, 95% CI [0.069, 0.196]) and high levels of competitive climate (B = 0.188, 95% CI [0.098, 0.288]) but not at the low level (B = 0.070, 95% CI [−0.008, 0.151]). Critically, the index of moderated mediation was 0.119, with a 95% CI of [0.004, 0.244] that excluded zero, formally indicating that the conditional indirect effects differed significantly across competitive climate levels. These results provide support for H5a. For autonomy-oriented help (H5b), the conditional indirect effects were significant at the mean (B = −0.036, 95% CI [−0.081, −0.001]) and high levels of competitive climate (B = −0.052, 95% CI [−0.113, −0.001]) but not at the low level (B = −0.020, 95% CI [−0.060, 0.002]). However, the index of moderated mediation for this pathway was −0.032, with a 95% CI of [−0.083, 0.003] that included zero, indicating that the conditional indirect effects did not differ significantly across competitive climate levels. H5b was therefore not supported. This pattern is consistent with the finding that status threat has a weaker and less robust direct association with autonomy-oriented help (B = −0.183, *p* < 0.05) than with dependency-oriented help (B = 0.460, *p* < 0.001), limiting the extent to which the competitive climate can systematically modulate this pathway.

Furthermore, to ensure the integrity of our structural model, we assessed the statistical stability of the focal paths after incorporating the interaction terms. The results indicate that the core relationships remained consistent; specifically, the positive effect of received task-related help on perceived status threat (B = 0.264, SE = 0.072, *p* < 0.001), the positive effect of perceived status threat on dependency-oriented help (B = 0.478, SE = 0.069, *p* < 0.001), and its negative impact on autonomy-oriented help (B = −0.184, SE = 0.079, *p* < 0.05) exhibited no substantial fluctuations. This consistency across different model specifications further reinforces the reliability of our findings. Nevertheless, while our primary hypothesized effects remained steady mathematically, we refrain from overstating their robustness, considering the inherent limitations of perceptually bound, self-report survey data.

#### 4.4.4. Robustness Checks

Because age and tenure were substantially correlated (r = 0.80, see Table 3), we examined variance inflation factors (VIF) to assess potential multicollinearity. The maximum VIF across all predictors was 2.81, well below the conventional threshold of 5, indicating that the shared variance between age and tenure does not unduly inflate standard errors or distort coefficient estimates. To further verify the stability of our results, we re-estimated the focal model twice: once excluding age (retaining tenure) and once excluding tenure (retaining age). The core coefficient (received task-related help → perceived status threat) remained significant in both specifications (without age: B = 0.195, *p* < 0.01; without tenure: B = 0.207, *p* < 0.01). These supplementary analyses confirm that the high age–tenure correlation does not substantively influence our conclusions.

## 5. Discussion

### 5.1. Interpretation of Main Effect Findings

Our empirical investigation, which is grounded in a two-wave time-lagged design, highlights the complex and inherently social-structural nature of post-help interaction. We found that receiving task-related help functions as a competence-threatening signal that predicts perceived status threats. Our findings suggest that status threat is associated with a bifurcated self-regulatory process in which recipients navigate subsequent interpersonal obligations. Specifically, status threat statistically accounts for the positive link between receiving help and dependency-oriented help, revealing a pattern of “defensive prosociality” while simultaneously exerting a suppressing effect on autonomy-oriented help.

### 5.2. Explanation of Mediation and Moderation Mechanisms

Our findings suggest that status threat is associated with a sophisticated psychological tension between two competing logics: social exchange and status-based self-regulation. On one hand, receiving task-related help activates the social exchange logic of reciprocity, where recipients feel a normative obligation to return the favor by providing high-quality, autonomy-oriented help to others. This explains the robust positive direct effect observed in our model. Importantly, we did not measure gratitude or perceived reciprocity obligations, so we cannot distinguish whether the positive direct path reflects grateful reciprocity, generalized reciprocity norms, relationship quality, or a learning orientation activated by receiving competent help. Our interpretation of this path as ‘consistent with reciprocity’ should be understood as one plausible interpretation among several. On the other hand, the concurrent activation of status-based anxiety is associated with a defensive self-regulatory response. In this logic, the recipient views the helping interaction through a competitive lens and prioritizes safeguarding their remaining competence advantage by withholding the very skills that would foster others’ independence. Whether the prosocial reciprocity or the defensive suppression dominates likely depends on the organizational climate. In environments where communal bonds are salient, the social exchange logic is expected to prevail. However, as our moderated mediation results suggest, in competitive settings, the status-defense mechanism becomes more salient, effectively throttling the impulse to provide autonomy-oriented help. This finding is characteristic of inconsistent mediation, wherein received help simultaneously activates two countervailing pathways: a positive direct pathway consistent with social exchange, and a negative indirect pathway through status threat that suppresses autonomy-oriented help.

## 6. Conclusions

### 6.1. Theoretical Implications

First, previous research on reciprocity has focused primarily on the binary distinction between “helping” versus “not helping”, associated with contradictory findings (some studies found positive reciprocity, while others found negative reactions). By distinguishing between dependency-oriented help and autonomy-oriented help as post-receipt responses, this study extends our understanding of reciprocity (Tai et al., 2023; Wang et al., 2023). Receiving help is associated with perceived status threat, which on one hand is associated with dependency-oriented help (a superficial form of “reciprocity”), and on the other hand is associated with a reduced tendency to provide autonomy-oriented help (a deeper form of capability transfer). This parallel mediation framework suggests that previous studies reached inconsistent conclusions because they failed to differentiate help types: when “reciprocity” is defined as dependency-oriented help, the effect is positive; when defined as autonomy-oriented help, the indirect effect is negative. Second, prior research has not yet clarified a plausible psychological mechanism underlying individuals’ behavioral shift from a cooperative orientation to a defensive orientation. We illuminate a novel status-defense mechanism by identifying perceived status threat as the “missing link” that explains the transition from communal cooperation to defensive self-regulation (DSR). Our finding that status threat exerts a suppressing effect on autonomy-oriented help is particularly crucial, as it reveals a more complex side of organizational citizenship: recipients may exhibit a diminished willingness to provide skill-transferring help, consistent with preserving their perceived relative competence. This suggests that the psychological reaction following help receipt is inherently defensive in nature, rather than a strategic hoarding of information. Third, among prior reciprocity studies, few have established competitive climate as a boundary condition. The present study introduces a competitive climate to further investigate the relationship between receiving task-related help and status threat. By identifying a competitive climate as a critical boundary condition, we clarify the contextual factors associated with this anxiety. Our findings provide evidence consistent with the hypothesis that the competence-threatening nature of help is not universal but contingent on environments that prioritize relative performance. This contextual pressure can magnify the stakes of routine interactions, rendering employees more sensitive to the competence-related implications of needing peer support in the workplace. By demonstrating that the indirect effect of receiving help is significantly more pronounced under high-competition conditions, our model provides a necessary boundary for the help-as-status perspective, showing how organizational structures shape individual psychological appraisal.

### 6.2. Practical Implications

While our empirical model focused on the psychological and behavioral mechanisms of post-help dynamics, our findings provide a foundation for several broader extrapolated implications for organizational management. First, this study finds that receiving task-related help potentially influences individuals’ perceived status threat, indicating that while helping behavior conveys support, it may also imply negative evaluations of the recipient’s competence. Therefore, managers should recognize that receiving help itself serves as a competence threat signal. Organizations should not rely solely on interpersonal assistance, but should instead focus more on cultivating employees’ self-efficacy and independence in problem-solving. For example, through regular training, experience sharing, and mentoring, organizations can help employees improve their professional competence and adaptability, thereby ensuring successful task completion while avoiding the status threat perception associated with over-reliance on help.

Second, this study distinguishes help into dependency-oriented help and autonomy-oriented help, demonstrating the importance of differentiating help types. Organizations need to encourage employees to provide more autonomy-oriented help while moderately intervening in dependency-oriented help. To better enhance overall organizational competitiveness and innovation capability, managers should encourage autonomy-oriented help and establish an incentive mechanism that values both team collaboration and individual growth.

Finally, this study finds that a competitive climate positively moderates the relationship between receiving task-related help and perceived status threat, indicating that managers should recognize the importance of competitive climate. Organizations need to prevent excessive competition from triggering employees’ negative psychological perceptions. Organizations can establish fair and transparent performance evaluation and promotion systems, clarify the boundaries of competition rules and cooperation, so that employees can pursue excellence in competition.

### 6.3. Limitations and Future Prospects

Despite these contributions, this study has several limitations. First, methodological limitations: Although Harman’s single-factor test indicated no serious common method bias (unexplained variance below 40%), all variables in this study were self-reported by the same respondents. This may be associated with perceptual inflation; that is, respondents may give high scores due to personal response styles, thereby overestimating the relationships among variables, particularly the relationships between receiving task-related help, competitive climate, and status threat. Furthermore, although the one-week time lag effectively separated the predictor and outcome variables, it may limit our ability to observe the enduring long-term effects of status threat on helping behavior. More importantly, our independent variable and mediator were measured concurrently at Time 1. Although theoretical logic suggests that the event of receiving help precedes the psychological appraisal of status threat, the simultaneous measurement limits our ability to definitively establish direction and fully rule out endogeneity. Because the independent variable and mediator were measured concurrently at Time 1, the temporal ordering assumed in our mediation model cannot be definitively established from the current data alone.

Second, sample and data limitations. The sample of this study was drawn solely from China, leaving the generalizability of our findings across different national and cultural contexts unclear. In addition, the sample was predominantly female (67%) and concentrated in the 20–40 age range (approximately 90%), which may further limit the generalizability of the findings to older or more gender-balanced workforces. Future research should replicate these findings in multi-country, multi-industry samples with greater demographic diversity. Moreover, data were collected only from help recipients, lacking helper-recipient dyadic data. Helpers and recipients may perceive the same helping event differently, which is precisely a critical aspect of understanding the helping interaction process.

Third, limitations of measured variables. This study did not examine the social status of helpers, nor did it measure the social distance between helpers and recipients. Help from helpers with different levels of status may have different effects on recipients, and varying social distances may also be associated with different intensities of status threat among recipients. Future research should directly measure gratitude and perceived reciprocity obligations to disentangle these competing explanations.

Fourth, the average variance extracted (AVE) for autonomy-oriented help (0.480) and competitive climate (0.486) fell slightly below the conventional 0.50 threshold, primarily due to items with relatively weaker loadings (e.g., autonomy-oriented help item 1, λ = 0.633; competitive climate item 3, λ = 0.647). While their robust composite reliability values (both > 0.80) provide acceptable support for convergent validity (Lam, 2012), future research should consider refining these items or adopting alternative scales.

To address the above limitations, future research should first employ longer longitudinal tracking, experimental designs, or daily diary methods with multiple intra-day measurement points to capture the precise chronological sequence from receiving help to the activation of status threat, thereby establishing relationships. Second, future research should collect samples from different countries and cultural contexts and adopt helper-recipient dyadic data to capture more objective behavioral signals than self-reports can provide. Third, future research should examine helper status and social distance between helpers and recipients, exploring how structural hierarchy alters the appraisal process of status threat, for example, whether help from a high-status supervisor mitigates threat through a developmental lens, whereas help from an equal-status peer directly is associated with status competition. Fourth, this study focused on task-related help; future research should contrast these dynamics with emotional help to determine whether affective support fosters social bonding rather than triggering status anxiety. Finally, the suppression effect on autonomy-oriented help warrants further examination through experimental designs to identify the specific cognitive barriers that prevent skill transfer.

## Figures and Tables

**Table 1:** Confirmatory factor analysis results.

Model	χ^2^	df	*p*	χ^2^/df	SRMR	CFI	TLI	RMSEA
Five-factor model	348.848	179	0.000	1.949	0.056	0.924	0.911	0.061
Four-factor model	594.412	183	0.000	3.248	0.110	0.816	0.789	0.094
Three-factor model	1049.594	186	0.000	5.643	0.140	0.615	0.565	0.136
Two-factor model	1438.376	188	0.000	7.651	0.168	0.442	0.377	0.162
One-factor model	1690.654	189	0.000	8.945	0.180	0.330	0.255	0.178

Note: The five-factor model includes receiving task-related help, perceived status threat, autonomy-oriented help provided, dependency-oriented help provided, and a competitive climate; the four-factor model combines receiving task-related help and perceived status threat as one factor, along with autonomy-oriented help provided, dependency-oriented help provided, and a competitive climate; the three-factor model combines receiving task-related help, perceived status threat, and autonomy-oriented help provided as one factor, along with dependency-oriented help provided and a competitive climate; and the two-factor model combines receiving task-related help, perceived status threat, autonomy-oriented help provided, and dependency-oriented help provided as one factor, along with a competitive climate.

**Table 2:** Factor loadings, AVE, and CR.

Variable	Factor Loading		AVE	CR
Receiving Task-Related Help	Receiving Task-Related Help 1	0.790	0.646	0.929
	Receiving Task-Related Help 2	0.858		
	Receiving Task-Related Help 3	0.805		
	Receiving Task-Related Help 4	0.758		
Perceived Status Threat	Perceived Status Threat 1	0.756	0.529	0.853
	Perceived Status Threat 2	0.726		
	Perceived Status Threat 3	0.698		
Providing dependency-oriented help	Providing dependency-oriented help 1	0.758	0.591	0.927
	Providing dependency-oriented help 2	0.767		
	Providing dependency-oriented help 3	0.761		
	Providing dependency-oriented help 4	0.804		
	Providing dependency-oriented help 5	0.752		
Providing autonomy-oriented help	Providing autonomy-oriented help 1	0.633	0.480	0.885
	Providing autonomy-oriented help 2	0.797		
	Providing autonomy-oriented help 3	0.731		
	Providing autonomy-oriented help 4	0.641		
	Providing autonomy-oriented help 5	0.647		
Competitive Climate	Competitive Climate 1	0.659	0.486	0.863
	Competitive Climate 2	0.674		
	Competitive Climate 3	0.647		
	Competitive Climate 4	0.799		

**Table 3:** Means, variances, and correlation coefficients of the variables.

Variable	M	SD	1	2	3	4	5	6	7	8	9	10
1. Age	31.490	7.828	—									
2. Gender	0.330	0.471	0.078	—								
3. Education	2.070	0.488	−0.096	−0.028	—							
4. Team Size	2.900	0.832	0.269 **	−0.042	−0.014	—						
5. Tenure	2.540	1.148	0.802 **	0.024	−0.072	0.317 **	—					
6. Receiving Task-Related Help	3.609	1.234	0.071	−0.018	−0.009	0.179 **	0.056	—				
7. Perceived Status Threat	2.291	0.826	−0.139 *	0.036	0.033	−0.081	−0.236 **	0.114	—			
8. Providing dependency-oriented help	4.240	1.067	−0.067	0.044	−0.069	−0.090	−0.113	0.125 *	0.387 **	—		
9. Providing autonomy-oriented help	5.160	0.805	0.032	−0.003	0.007	−0.012	0.038	0.337 **	−0.084	−0.092	—	
10. Competitive Climate	3.270	1.093	−0.070	0.016	−0.117	−0.020	−0.118	−0.132 *	0.293 ***	0.200 **	−0.032	—

Note: The reported correlation coefficients are Pearson’s correlation coefficients. *** indicates *p* < 0.001, ** indicates *p* < 0.01, and * indicates *p* < 0.05.

**Table 4:** Path analysis results for perceived status threat.

Variable	Perceived Status Threat			
	Model 1		Model 2	
	B	SE	B	SE
Control Variables				
Age	0.152	0.117	0.140	0.116
Gender	0.040	0.070	0.040	0.069
Education	0.021	0.070	0.024	0.069
Team Size	−0.009	0.073	−0.036	0.073
Tenure	−0.391 **	0.115	−0.379 **	0.129
Independent Variable				
Receiving Task-Related Help			0.163 *	0.075
R^2^	0.085		0.115	
ΔR^2^	—		0.03	
Adjusted R^2^	0.067		0.094	
F	5.760 ***		6.419 ***	

Note: *** indicates *p* < 0.001, ** indicates *p* < 0.01, and * indicates *p* < 0.05.

**Table 5:** Path analysis results for providing dependency-oriented help.

Variable	Providing Dependency-Oriented Help							
	Model 1		Model 2		Model 3		Model 4	
	B	SE	B	SE	B	SE	B	SE
Control Variables								
Age	0.058	0.112	0.047	0.111	0.004	0.104	−0.015	0.104
Gender	0.043	0.067	0.044	0.066	0.029	0.062	0.025	0.062
Education	−0.080	0.067	−0.080	0.066	−0.089	0.062	−0.090	0.062
Team Size	−0.063	0.070	−0.087	0.070	−0.062	0.065	−0.070	0.066
Tenure	−0.142	0.113	−0.135	0.112	−0.012	0.107	−0.030	0.109
Independent Variable								
Receiving Task-Related Help			0.154 *	0.070			0.078	0.069
Mediating Variable								
Perceived Status Threat					0.471 ***	0.065	0.460 ***	0.069
R^2^	0.026		0.056		0.235		0.244	
ΔR^2^	—		0.030		0.179		0.009	
Adjusted R^2^	0.006		0.033		0.216		0.222	
F	1.665		2.931 **		15.175 ***		13.233 ***	

Note: *** indicates *p* < 0.001, ** indicates *p* < 0.01, and * indicates *p* < 0.05.

**Table 6:** Bootstrap test results for the mediation effects of perceived status threat.

Effect Type.	B	Bootstrap 95% CI	
		Lower Bound	Upper Bound
Indirect Effect	0.081	0.008	0.179
Direct Effect	0.078	−0.057	0.208

Note: Number of resamples = 5000.

**Table 7:** Path analysis results for providing autonomy-oriented help.

Variable	Providing Autonomy-Oriented Help							
	Model 1		Model 2		Model 3		Model 4	
	B	SE	B	SE	B	SE	B	SE
Control Variables								
Age	0.006	0.116	−0.018	0.109	0.016	0.116	0.007	0.109
Gender	−0.007	0.070	−0.005	0.065	−0.004	0.069	0.003	0.065
Education	−0.006	0.069	−0.004	0.065	−0.003	0.069	0.001	0.065
Team Size	−0.035	0.073	−0.097	0.069	−0.035	0.073	−0.105	0.068
Tenure	0.060	0.117	0.076	0.110	0.033	0.120	0.006	0.113
Independent Variable								
Receiving Task-Related Help			0.390 ***	0.065			0.423 ***	0.065
Mediating Variable								
Perceived Status Threat					−0.092	0.080	−0.183 *	0.079
R^2^	0.026		0.162		0.031		0.193	
ΔR^2^	—		0.136		−0.131		0.162	
Adjusted R^2^	0.001		0.136		0.001		0.164	
F	1.315		7.578 ***		1.254		7.773 ***	

Note: *** indicates *p* < 0.001 and * indicates *p* < 0.05.

**Table 8:** Bootstrap test results for the suppression effects of perceived status threat.

Effect Type	B	Bootstrap 95% CI	
		Lower Bound	Upper Bound
Indirect Effect	−0.031	−0.100	−0.001
Direct Effect	0.423	0.290	0.538

Note: Number of resamples = 5000.

**Table 9:** Path analysis results for a competitive climate.

Variable	Perceived Status Threat							
	Model 1		Model 2		Model 3		Model 4	
	B	SE	B	SE	B	SE	B	SE
Control Variables								
Age	0.152	0.117	0.140	0.116	0.122	0.110	0.105	0.109
Gender	0.040	0.070	0.040	0.069	0.039	0.066	0.035	0.065
Education	0.021	0.070	0.024	0.069	0.068	0.066	0.075	0.065
Team Size	−0.009	0.073	−0.036	0.073	−0.057	0.070	−0.064	0.069
Tenure	−0.391 **	0.115	−0.379 **	0.129	−0.320 **	0.111	−0.304	0.111
Independent Variable								
Receiving Task-Related Help			0.163 *	0.075	0.224 **	0.072	0.256 ***	0.073
Moderating Variable								
Competitive Climate					0.408 ***	0.072	0.412 ***	0.071
Interaction Term								
Receiving Task-Related Help × Competitive Climate							0.135 *	0.069
R^2^	0.085		0.115		0.263		0.298	
ΔR^2^	—		0.030		0.196		0.035	
Adjusted R^2^	0.067		0.094		0.242		0.275	
F	5.760 ***		6.419 ***		14.631 ***		14.858 ***	

Note: *** indicates *p* < 0.001, ** indicates *p* < 0.01, and * indicates *p* < 0.05.

**Table 10:** Moderated mediation of Competitive Climate on Indirect Effects via Perceived Status Threat.

Conditional Indirect Effect	Dependency-Oriented Help		Autonomy-Oriented Help	
	B	Bootstrap 95% CI	B	Bootstrap 95% CI
Low Competitive Climate (−1 SD)	0.070	[−0.008, 0.151]	−0.020	[−0.060, 0.002]
Mean Competitive Climate	0.129	[0.069, 0.196]	−0.036	[−0.081, −0.001]
High Competitive Climate (+1 SD)	0.188	[0.098, 0.288]	−0.052	[−0.113, −0.001]
Index of Moderated Mediation	0.119	[0.004, 0.244]	−0.032	[−0.083, 0.003]

Note: A highly competitive climate represents one standard deviation above the mean, and a less competitive climate represents one standard deviation below the mean. The number of resamples = 5000.

## Data Availability

The data presented in this study are available on request from the corresponding author.

## Appendix A. Measurement Items

**Received Task-Related Help** (Time 1; 1 = very infrequent, 7 = very frequent)

*Please recall your actual work situation over the past week and indicate the frequency of the following events*:

1. Someone took the initiative to assist me with difficult tasks without my request.
2. Someone actively shared my excess workload.
3. Someone took on additional work responsibilities to help me solve problems.
4. Someone supported me when I fell behind in my work progress.

**Perceived Status Threat** (Time 1; 1 = strongly disagree, 7 = strongly agree)

*Please recall the situations where you received help, and indicate the extent to which you agree with the following statements:*

1. I felt that my status within the team was damaged.
2. I felt it was difficult to establish a foothold in the team.
3. I felt that I was not respected in the team.

**Competitive Climate** (Time 1; 1 = strongly disagree, 7 = strongly agree)

*Please evaluate the following statements based on the daily work situation in your team*:

1. My supervisor frequently compares me with other team members.
2. The degree of recognition I receive depends on how my performance compares to that of other members.
3. I feel that everyone in our team wants to be at the top.
4. My team members frequently compare their performance results with mine.

**Autonomy-Oriented Help** (Time 2; 1 = very infrequent, 7 = very frequent)

*Please evaluate the following statements based on your recent work interactions*:

1. When others encountered difficulties, I helped them understand the crux of the problem so they could solve similar problems themselves in the future.
2. When others encountered difficulties, I helped them learn how to solve the problem on their own.
3. When others encountered problems, I provided new perspectives so they could solve similar problems themselves later.
4. When others encountered problems, I shared my experience with them so they could handle similar situations themselves in the future.
5. I guided others to think about work-related problems so they could handle similar problems themselves in the future.

**Dependency-Oriented Help** (Time 2; 1 = very infrequent, 7 = very frequent)

*Please evaluate the following statements based on your recent work interactions*:

1. When others encountered difficulties, I directly solved the problem for them without teaching them the relevant skills.
2. I directly helped others solve problems without caring whether they learned how to solve similar issues.
3. When others encountered difficulties, I directly gave them the solution.
4. When others encountered difficulties, I directly solved the problem for them.
5. When others encountered difficulties, I directly solved the problem for them, even though they might ask for my help again for similar problems in the future.
